# Reducing harm and promoting positive media use strategies: new perspectives in understanding the impact of preschooler media use on health and development

**DOI:** 10.1186/s41155-023-00262-2

**Published:** 2023-08-09

**Authors:** Caroline Fitzpatrick, Marie-Andrée Binet, Emma Cristini, Maíra Lopes Almeida, Mathieu Bégin, Giana Bitencourt Frizzo

**Affiliations:** 1 Département de l‘enseignement au préscolaire et au primaire, Université de Sherbrooke, Sherbrooke, Québec Canada; 2 Department of Childhood Education, University Johannesburg, Johannesburg, South Africa; 3 Faculté de médecine et des sciences de la santé, Université de Sherbrooke, Sherbrooke, Canada; 4 Department of Psychology, Universidade Federal Do Rio Grande Do Sul, Porto Alegre, Brazil

**Keywords:** Screen time, Screen media, Media content, Infant, Preschooler, Development, Harm-reduction

## Abstract

Most children grow up in homes with easy access to multiple screens. Screen use by children between the ages of 0 to 5 has become a worldwide preoccupation. In the present narrative review, we examine child and parent screen use and its contribution to physical, cognitive, and social developmental outcomes. As research has mostly focused on the adverse consequences of screen media, we aim to depict both the negative and the positive influences of screen usage. To provide a more nuanced portrait of the potential benefits and harms of screen use, we examine how consequences of media use vary according to the content of media (ex., educational, violent), context (ex., using screens during mealtimes), and the nature (ex., passive vs active use) of child screen use. Our review supports existing screen time guidelines and recommendations and suggests that media content, the context of use, and the nature of child use, as well as the parent’s own screen use, be considered clinically. Future research should seek to clarify how these dimensions jointly contribute to child screen use profiles and associated consequences. Finally, child sex, behavioral/temperamental difficulties, and family adversity appear to contribute to child screen use and its consequences and should be considered in future research. Suggestions for harm-reduction approaches are discussed.

## Introduction

In the context of increasingly and widely available screen media use by children, the aim of the present narrative review is to present an overview of the main positive and negative consequences of preschooler’s screen use (Radesky et al., 2015a, 2015b). We also propose a wider conceptualization of child screen use that moves beyond a single focus on *screen time* to include additional dimensions of child screen use including contents, as well as nature and context of use. We will consider how the consequences of screen use by children and parents may vary according to these additional dimensions and according to individual child characteristics. Finally, we will explore possible mechanisms that may account for different media use consequences and offer harm reduction as a useful framework for clinical practice.

## Background

### The media landscape of young children

Children’s digital landscape has changed dramatically over the last decades. For one, the popularity of portable device use has risen sharply since their market introduction in 2010. For instance, 95% of children between the ages of 0 and 8 now have access to a smartphone and 78% have access to a tablet. Nearly 1 in 2 children also possess their own tablet (Rideout & Robb, 2020). Although viewing traditional television programs and movies remains popular, the popularity of YouTube continues to climb among young and older children. Finally, the majority of households subscribe to Netflix, Disney + , or other streaming services which can further multiply viewing occasions (Bleakley et al., 2014; Rideout & Robb, 2020). The changing landscape has had an impact on screen use by very young children as well. According to an American survey, children under the age of 2, spend an average of 49 min per day with screens (Rideout & Robb, 2020).

Parents are also important consumers of digital media. Before the COVID-19 crisis, parents of preschoolers reported an average of 4 h of screen media use per day (Bleakley et al., 2014) and more recently, mothers of 3-year-olds self-reported an average of 3 h per day of screen media use (Madigan et al., 2020a, 2020b). According to one Canadian study, 93% of adults of child-rearing age (ages of 18 to 44), are heavy users of the Internet (Académie de la transformation numérique, 2022). Furthermore, in another Canadian study conducted during the pandemic, parents reported spending an average of 6.35 h per day engaged with screen media (Fitzpatrick, et al., 2022a, 2022b).

Parent media use may influence child development through increased *technoference*, which refers to the interference of technology in parent–child relationships (McDaniel & Radesky, 2018). In particular, technoference can affect parenting quality by displacing the amount of time parents are available and attentive to their children (McDaniel & Radesky, 2018). There is evidence that increased screen time can distract parents from parent–child interactions which can then lead to parents being less responsive and sensitive to their children (Braune-Krickau et al., 2021; Kildare & Middlemiss, 2017; Konrad et al., 2021; Ochoa et al., 2021).

Finally, parental media use may influence child outcomes through indirect exposure to media. A survey indicated that 39% of US children under age 8 live in homes where a television is on all or most of the time, even if no one is watching (Rideout & Robb, 2020). A study conducted by Lapierre et al. (2012) highlighted that toddlers (less than 24 months) are exposed to approximately 5.5 h of background television per day as opposed to 3.5 h for older children (up to 8 years). Furthermore, according to a study by Masur et al. (2016), 69% of parents keep the television turned on all or half of their child’s waking time.

### Guidelines and recommendations

In the midst of the increased digitization of children’s lives, the World Health Organization (WHO, 2019) and the American Academy of Pediatrics (Hill et al., 2016) recommend limiting preschooler screen time to 1 and 2 h daily. They also recommend that children younger than 18 months not be exposed to any screen time. Other organizations preoccupied with the health and wellbeing of children have formulated similar recommendations (ex., Canadian Pediatric Society, 2022). According to a meta-analysis conducted prior to the pandemic, only 1/4 of children younger than 2 and 1/3 of children between the ages of 2 and 5 meet pediatric healthy screen time guidelines internationally (McArthur et al., 2022a, 2022b).

### Theoretical and practical considerations

Child screen media use and its consequences can be understood within an *ecosystemic framework*, in which screen time is conceptualized as being influenced by children’s individual characteristics, the various settings (ex., home, neighborhood) in which they interact (Bronfenbrenner & Morris, 2007), and the larger socio-historical context in which children find themselves. For instance, the COVID-19 pandemic represents an important historical event which resulted in increased screen intake for children between the ages of 0 and 18 (Hartshorne et al., 2021; Moore et al., 2020).

Similarly, biopsychosocial approaches, according to which development is shaped by the experience of risk and protective factors at individual, family, and community levels (Wade & Halligan, 2017) also shed light on child and family media use and its consequences. For example, more difficult children may elicit more screen time because it is more challenging for parents to engage them in non-screen-based activities. In addition, parents’ access to personal and material resources (ex., media literacy, childcare, mental health) may enhance the positive and buffer against the negative consequences of preschooler media use. Lower socioeconomic status children and visible minority children also spend more time with screen media, despite being less likely than higher socioeconomic families to own a computer (Rideout & Robb, 2020). As such, we seek to review individual and family characteristics that may contribute to child screen media use and its consequences.

On a practical level, it is important to acknowledge that research examining *screen time*, referring to the duration of screen use, has ruled the literature. Although the amount of time children accumulate in front of screens is important to consider, a sole focus on duration of use can obscure how the *content*, *context*, and *nature* of child screen use may contribute to child outcomes. Screen content refers to the extent to which programs, movies and apps, are educational, age-appropriate, prosocial, or violent. Furthermore, the general pacing or the speed at which action unfolds in programs and movies is an important feature of content (Christakis et al., 2018). Similarly, the nature of children’s media use, for instance whether the use is interactive or passive, is also likely to influence its impact. Finally, the context of screen use including timing (ex., use right before bedtime, at dinner time), location (ex., bedroom media use), and parental motifs for using media with their child (i.e., to keep their child busy or for educational purpose) are also likely to contribute to its consequences on children.

Using a more nuanced and comprehensive approach to understanding screen use may be especially important for increasing our ability to understand which patterns or profiles of use are most closely associated with positive, negative, or neutral consequences in children. Applying this perspective to child and parent screen habits is in line with clinically effective, harm-reduction, and positive digital media use approaches. Harm-reduction approaches recognize that some problematic behaviors, such as screen use are unavoidable and thus seek to minimize the risks of these behaviors on health outcomes (Leslie, 2008). Positive digital media use strategies involve identifying the circumstances under which digital media use is favorable to health and development. Since most parents struggle to adhere to screen time recommendations, using a more nuanced approach to screen use may be especially useful for identifying uses that are related to positive and negative child outcomes.

In the following section, we overview evidence linking infant/toddler and preschooler screen time duration, content, nature, and context of use to negative and positive child outcomes. We also present research examining the negative and positive outcomes of parent screen use. Finally, we explore how individual differences may mitigate these associations.

### Child media use

#### Negative consequences: infants and toddlers (0–2)

##### Duration

Because of the importance of caregiver-infant interactions for brain growth and development at this age, pediatricians recommend no screen time between 0 and 2. There is evidence that infant and toddler exposure to screen time contributes to reduced sleep quality, including shorter sleep duration, and more irregular nap and bedtimes (Cespedes et al., 2014; Chen et al., 2019; Vijakkhana et al., 2015). There is also evidence that greater hours of television viewing between 0 and 3 forecasts worse reading and working memory skills by the time children enter school (Zimmerman & Christakis, 2005). In addition, the duration of exposure to screens at the age of 6 months has been linked to lower cognitive and language development at 14 months (Tomopoulos et al., 2010). Finally, research employing electroencephalography has shown that screen time at 12 months is linked to altered brain activity before age 2 years (Law et al., 2023). These changes in brain activity were then found to mediate associations between infant screen time and worse executive functions.

##### Content

Some studies have simultaneously examined the duration and content of young children’s screen use. For instance, toddlers exposed more frequently to adult-directed content developed worse executive function skills (Barr et al., 2010). According to another study, the duration of screen use and exposure to content designed for older children and adults is linked to lower cognitive and language development at 14 months (Tomopoulos et al., 2010). In contrast, other research has found that exposure to child- but not adult-directed media can be detrimental to infants and toddlers’ language development (Duch et al., 2013). Finally, research indicates that the duration and content of screen exposure at 21 months, in particular exposure to noneducational child media, such as cartoons, is associated with an increased risk of developing externalizing behaviors at 33 months (Tomopoulos et al., 2007).

##### Nature of use

There is less research addressing how the nature of infant screen use can influence their health and development. One exception is a study of 715 UK infants and toddlers, showing that the use of touch screen devices can interfere with sleep quality (Cheung et al., 2017). Touch screen device use may undermine sleep through increased exposure to blue light or because touch screens may lead to increased arousal in young children, which can then be detrimental to sleep.

##### Context

The timing and location of an infant’s screen use are also important to consider. According to a study with Thai toddlers, mealtime screen use contributes to higher calorie intake and prolonged eating duration. Mealtime screen use by toddlers was also associated with a greater risk of having a BMI in the obese range (Teekavanich et al., 2022). It is estimated that 60% of parents in the UK and Singapore expose children to mealtime screen use (Goh & Jacob, 2012; Wright et al., 2007). Other research suggests that using screens as means to regulate toddler behavior may have a negative impact on their development of self-regulation. For instance, if a child is throwing a tantrum in a public space, parents may hand the child a portable device to help distract them from their experience of negative emotions (Cardoso Azevedo et al., 2022; Radesky et al., 2016a, 2016b). The use of screen-based emotion regulation strategies by parents has been linked to the development of more *problematic media use* (ex., loss of interest in activities that don’t involve screens) in toddlers (Coyne et al., 2021).

#### Negative consequences: preschoolers (2–5 years of age)

##### Duration

Research has linked preschooler screen time to adverse physical, cognitive, and psychosocial outcomes. Studies have linked preschooler screen time to poor sleep quality (Beyens & Nathanson, 2019; Helm & Spencer, 2019; Hiltunen et al., 2021; Zhu et al., 2020). In addition, longitudinal research based on a Canadian population-based birth cohort has found that screen time between the ages of 2 and 4 forecasts higher BMI, waist circumference, reduced fitness, and lower levels of physical activity involvement in later childhood (Fitzpatrick et al., 2012; Pagani, et al., 2010). There is also convincing research indicating that excessive screen time by preschoolers can increase their risk of experiencing developmental delays across motor, psychosocial, cognitive, and language developmental domains (Madigan et al., 2019; McArthur et al., 2022a, 2022b). Furthermore, recent evidence suggests that preschooler screen intake is prospectively linked to reduced attention skills, effortful control, and emotional regulation skills (Fitzpatrick et al, 2023, 2022a, 2022b; Tamana et al., 2019). Finally, in one study researchers measured screen time, along with other features of preschooler screen habits (ex., exposure to violent and fast-paced content, using screens without parental supervision) and found that when considered together, worse screen habits (ex., exceeding the time recommendations, exposure to lower quality content, viewing along) were associated with lower microstructural integrity in the white matter brain tracts that support early language development (Hutton et al., 2020).

##### Content

One of the most studied features of content is the impact of exposure to scenes of violence. According to an experimental study, children of parents who received a harm-reduction intervention to help them replace violent contents in their child’s *media diets*, with educational or prosocial contents, showed more social competence and less externalizing behavior 6 months later, relative to control children (Christakis et al., 2013). Other experimental research has found that preschooler exposure to violent content (ex., Mighty Morphin’ Power Rangers) but not exposure to non-violent content (ex., *Mister Rogers*) can have a negative impact on child concentration (Geist & Gibon, 2000). Longitudinal research conducted in community settings supports these results. As an example, in a study of 1800 Canadian children, parents reported exposure to violent media when their child was 4. Teachers then reported academic and behavioral adjustments when the children were 8 and 13 years old. Results indicated that exposure to scenes of violence was associated with later increased risk of poor attention, emotional distress, and antisocial behavior (Fitzpatrick et al., 2012; Pagani et al., 2023). These correlational associations were adjusted for child baseline aggressive and impulsive behavior, parent antisocial behavior, hostile parenting, and family income.

Other research suggests that the pacing and realism of programs are important features of content (Christakis, 2011; Lillard et al., 2015). Fast-paced programs that involve quick scene changes and frequent camera cuts are effective in sustaining child attention without soliciting cognitive effort (Christakis et al., 2018). This type of media content is therefore likely to undermine and disrupt the development of sustained attention and executive function skills in young children. Experimental research has also found that young children’s exposure to content that is unrealistic or fantastical (ex., a human superhero that can fly) can deplete their executive functions in the short term (Lillard et al., 2015). This is believed to be the case because this type of content contradicts children’s basic understanding of the world (Smith, 2020).

Researchers have also been concerned with the impact of childhood exposure to advertisements. In Quebec, Canada as well as in Brazil, laws prohibit advertising to children under the age of 13. In Canada, this law was designed to prevent the promotion of sugary cereals or fast-food chains towards children. Banning advertising directed at children is a measure considered effective by researchers in reducing the consumption of sugary foods (Bergeron & Paquette, 2014). In the context of rising childhood obesity rates in Canada and internationally, researchers argue that this regulation could benefit the health of children worldwide (Potvin Kent et al., 2019).

Finally, insufficient research has examined other features of children’s media content such as the extent to which media contains racial and/or gender stereotypes or lacks positive representations of diversity. Gender and racial stereotypes are common in media marketed at older school-aged children and adolescents and have been shown to impact the behavior, self-esteem, body image, and wellbeing of viewers (Adams-Bass, et al., 2014; Anderson et al., 2010; Coyne et al., 2016; Gerding et al., 2014). Less is known about how these contents may influence younger children.

##### Nature of use

Screens that require users to employ touchscreen technology or those designed for individual use (i.e., smartphones vs TVs) have been linked to reduced theory of mind ability in preschoolers (Konok et al., 2021). Other studies on touchscreen devices have found adverse impacts on fine motor skills, effortful control, and internalized/externalized behaviors (Lin et al., 2017, 2020; Nathanson & Beyens, 2018; Webster et al., 2019). In general, research has proposed that passive uses (ex., viewing television shows), compared to more active uses (ex., using a computer), are especially linked to negative child health and developmental outcomes.

##### Context

Mealtime, bedroom, and bedtime screen use can also influence preschooler health and development. Research has found that mealtime television viewing by preschoolers is associated with a lower intake of fruits and vegetables and more intake of obesogenic foods like sugar-sweetened drinks and fast food (Wenhold & Harrison, 2018). Preschooler exposure to TV during family meals has also been linked to lower language scores (Martinot et al., 2021). According to a Canadian study, preschoolers who grow up with a television in their bedroom have greater BMIs, less healthy eating habits, and experience more emotional distress and victimization by age 12 (Pagani et al., 2019). Children with access to bedroom screens also appear to face greater health and psychosocial risks through increased access to screens and greater exposure to violent content (Gentile et al., 2017). Finally, research also indicates that bedtime screen use is associated with reduced sleep quality as well as reduced theory of mind ability (Beyens & Nathanson, 2019; Nathanson & Fries, 2014; Staples et al., 2021).

### Positive consequences of child media use

#### Infants and toddlers

##### Screen time

There have been few documented benefits of screen time exposure on very young children. More specifically, research has identified a *video-deficit effect*, in which experimental studies consistently show the advantages of real-world interactions over similar content presented via a screen for child learning (Barr, 2010).

##### Content and context

Research which has identified positive outcomes of infant and toddler screen use has simultaneously considered content and context. Findings based on these dimensions are presented together. To date, there are no scientific studies supporting the educational benefits of the so-called educational programs such as *Baby Einstein* for toddler development. Researchers agree that such products can have a positive effect only under circumstances in which contents are viewed repeatedly and viewing is accompanied by parents commenting on the contents (Nichols, 2022). As such, it would appear that the accompanying interactions, rather than the quality of these contents are responsible for any educational gains. Indeed, the presence of a competent co-viewer appears to boost babies’ language learning from screen media, much in the same way parents facilitate language learning in live interactions (Linebarger & Vaala, 2010). Other research has confirmed these findings suggesting that mother-infant interaction has a strong effect on video learning (Zack & Barr, 2016). Similarly, experimental research has found that children who are exposed to a new word repeatedly through the viewing of a DVD are no better at learning the new word than control children not exposed to the DVD (DeLoache et al., 2010). According to the same study, parents have a tendency to overestimate infants’ learning through videos.

##### Nature of use

Finally, the nature of some screen use activities may offer benefits to infants and toddlers. The use of video chat technologies (ex., Skype, Facetime) that enable interactive, two-way, live exchanges between children and adults can facilitate language learning (Roseberry et al., 2014). In addition, the use of video chat technologies can foster social benefits by helping maintain family connections with parents that are physically absent (McClure & Barr, 2017; McClure et al., 2015). Recent research taking place during the pandemic found that videochat allowed sensitive interactions between children and grandparents and favored child engagement and positive affect (Roche et al., 2022). Indeed, most health organizations deem this type of activity safe even in the early years. Finally, the use of electronic books or eBooks, with infants and toddlers appears as a promising strategy for providing children with access to a wide range of children’s literature in multiple languages through widely available tablets and electronic readers (Tomopoulos et al., 2019). This could also increase family access to culturally appropriate reading materials that are easily accessible at low cost (Tomopoulos et al., 2019). Unfortunately, current research suggests that both toddlers and parents verbalize less during eBook reading than during traditional book reading (Munzer et al., 2019). According to a review of research, eBook reading offers less educational benefits than reading traditional books (Reich et al., 2016). Since young children are likely to benefit more from face-to-face interactions, future research could help to clarify if increasing interactions during ebook reading may lead to improved learning outcomes. Alternatively, research could clarify the presence of generational effects. For instance, it may be the case that *digital native* parents are more likely to engage children in face-to-face interactions during eBook reading than non-digital native parents.

#### Preschoolers

##### Screen time and content

There are documented positive content-dependent outcomes of preschoolers viewing of high-quality educational media. For instance, exposure to media content that is age-appropriate and educational (ex., Sesame Street) has been linked to improved cognitive outcomes in preschool-aged children (Anderson et al, 2001). Exposure to non-violent, educational content has been linked to improved preschooler social competence (Christakis et al., 2013). Furthermore, according to another experimental study, 2- and 3-year-olds were more likely to exhibit self-control (e.g., waiting before unwrapping a gift bag) after playing an educational app than after watching a cartoon. In the same study, preschoolers also scored higher on an assessment of working memory after playing the educational app (Huber et al., 2018).

##### Context

The social context in which children use screens is also related to its outcomes. Coviewing screen media with a caregiver has been linked to positive cognitive development in a population-based sample of Korean preschoolers (Lee et al., 2017). Furthermore, according to a meta-analysis of 42 studies, parental co-viewing of screens with preschoolers is associated with better language outcomes, especially in boys. Parental joint media engagement with their child, which can involve engaging their child in interactions centered around screen content, is also associated with better preschooler language development (Sundqvist et al., 2021).

##### Nature of use

Much like with younger children, highly interactive videochat technology use is likely to provide social benefits to older preschool-aged children. Recent research suggests that preschoolers can learn similarly from live and videochat book reading since both conditions allow contingent and sensitive interactions (Gaudreau et al., 2020). Indeed, a study with 2- and 3-year-olds found that videochat elicited as much participation from children in the form of imitating actions and responding to their interaction partner as a live interaction (Myers et al., 2019).

### Parent media use

#### Negative consequences

##### Infants and toddlers

Parental technoference has been studied by using a *still face* paradigm where parent phone use is substituted for the still face. Consistent with the standard still-face experiments, infants displayed increased negative affect, decreased positive affect, and increased bids for parental attention when parents were engaged with their cell phones (Myruski et al., 2018). Similarly, in a German American replication, parents of toddlers were instructed to respond to a text on their mobile phones and complete a paper-based questionnaire during a parent–child interaction (Konrad et al., 2021). Results indicated that responding to the text message and completing the questionnaire both led mothers to be less responsive and initiate fewer activities with their child. Another experiment evaluated toddlers’ vocabulary acquisition during parent–child interaction with or without interruptions by their parent’s smartphones. Children learned the new word only in the condition with no interruption, suggesting that parental technoference may impair children’s learning outcomes (Reed et al., 2017).

##### Preschoolers

There is also evidence of the negative effects of parental technoference on preschoolers. Research has demonstrated associations between parent mobile device use and fewer parent–child verbal and non-verbal interactions (Radesky et al., 2015a, 2015b). Parental media use and technoference have also been related to more externalizing behavior symptoms, negative emotional responses, and self-regulation difficulties in children (Choe et al., 2022; McDaniel & Radesky, 2018). Moreover, parent screen use is likely to shape preschoolers’ later screen use habits (Lee et al., 2022). For instance, parent screen time is associated with more digital media intake among 5- to 6-year-olds (Jago et al., 2014). Parents’ media use and family use during mealtime also contributed to preschooler screen time (Birken et al., 2011). In this sense, parent screen time may be a key element in children’s family media ecology (Lauricella et al., 2015).

#### Background media

In most studies, exposure to screens is considered as a primary activity. However, as previously noted, young children are often exposed to screens indirectly, in the form of background screen exposure. As infants and toddlers are sensory-oriented, sounds and movements can disrupt their ongoing activity (Gillioz et al., 2022; Kirkorian et al., 2009). This can be the case if the child is playing or eating, and the television is on in the background. An important deleterious effect of this type of exposure would be on the child’s attentional focus during playtime (Schmidt et al., 2008; Setliff & Courage, 2011). In particular, background media exposure is believed to periodically draw the child’s attention away from ongoing play activities resulting in less complex or lower-quality play. Background media exposure has also been linked to poorer language development. Language acquisition and development in early childhood are strongly associated with parental language input. Experimental research has found that adult-directed background television reduces the quantity and quality of their interactions with toddlers (Kirkorian et al., 2009). In addition, according to another study, preschoolers growing up in homes where the television is *always on* watched more television and read less than other children (Vanderwater et al., 2005).

#### Positive consequences

Parents report several benefits of their own screen use. For one, parental screen use may help them feel connected to others and access social support (Coyne et al., 2022a, 2022b; Haslam et al., 2017). Parents also report using social media as a source of information, advice, and emotional support from other parents in a time-efficient manner (Haslam et al., 2017). Findings from another study with American mothers showed that media allowed them to connect with family members and that it helped alleviate some of the challenges of motherhood (Coyne et al., 2022a, 2022b). Parents also report that mobile device use helps them reduce feelings of stress (Radesky et al., 2016a, 2016b). Mobile phone use also helps parents *mentally escape* during more negative or boring child-rearing activities. Lastly, intervention research suggests that the targeted use of text messages sent to parents to reinforce messages provided in an informational booklet can improve family media habits (Wen et al., 2020). In particular, using parents’ mobile phones to share information was found to reduce infant screen time and increase beneficial tummy time.

#### Viewer characteristics and individual differences/vulnerability factors

Differential susceptibility and stress-diathesis models predict that individuals are likely to vary in their level of sensitivity to environmental stressors and influences (Valkenburg & Peter, 2013). As such, it remains important to better understand the role of individual differences in the development of child media habits and their consequences. Here, we review the literature on viewer characteristics including child age, sex and gender, behavioral and temperamental profile, ethnicity and culture, and family context.

##### Child age

Research has found that an early-age introduction to screen media in combination with high levels of screen time and the absence of interactions with caregivers during usage is associated with less-than-optimal brain and cognitive development (Hutton et al., 2020; Supanitayanon et al., 2020).

Younger children may be more sensitive to time spent in front of screens, particularly to displaced time for key sensorimotor experiences and for environment-dependent brain growth and development. A younger age of onset of screen use has been linked to child screen use trajectories. For instance, research has found that early introduction to screens (around 2 or 4 months) is linked to greater screen intake at age 2 (Hish et al., 2020). Furthermore, as children age, they tend to spend increasing amounts of time using screens (Hoyos Cillero & Jago, 2010; Krogh et al., 2021).

##### Sex and gender

Boys appear to be more vulnerable to the negative effects of screen use on externalizing behavior problems and language skills (Pagani et al., 2023; Gentile, et al., 2017; Liu et al., 2021; MacGowan & Schmidt, 2021). A recent meta-analysis also reported that boys tended to benefit more from co-viewing with parents than girls (Madigan et al., 2020a, 2020b). In contrast, other studies have found no influence of sex on associations between preschoolers’ screen time and school readiness, social-emotional health, or self-regulation skills (Cliff et al., 2018; Neville et al., 2021; Vanderloo et al., 2022). Less research has examined the role of gender in child screen use and its consequences. Screen media use by boys and girls may influence their gender role development through exposure to contents that feature stereotypically feminine or masculine characters (Coyne et al., 2016). Furthermore, it is also possible that child gender contributes to exposure to different types of media content by influencing their preferences for violent or prosocial contents. Finally, child gender roles could also moderate associations between exposures to certain types of contents (ex., violence, gender stereotypes) and their consequences.

##### Temperament/behavioral profile

Research on infants has shown that children with poor self-regulation at 9 months are likely to be exposed to more screen time at age 2 (Radesky et al., 2014). Children’s general dispositions towards pleasure-seeking behavior (surgency) and proneness to negative emotions such as anger and frustration (negative affectivity) have also been found to contribute to child media use habits (McArthur et al., 2022a, 2022b). Furthermore, associations between child temperament and screen use appear to be more pronounced in families facing greater levels of social risk (McArthur et al., 2022a, 2022b). Toddlers with more feeding difficulties (ex., being a picky eater, refusing food) have also been shown to be at greater risk of mealtime screen exposure (Teekavanich et al., 2022). The effects of violent contents on aggression also appear to be amplified for children and adults showing higher levels of aggression (Bushman, 1995; Josephson, 1987), though research on young children remains sparse. Last, certain child psychopathologies are associated with greater screen intake. According to one study, 3-year-olds with more severe ADHD symptoms, but not children with ASD symptoms, were likely to spend more time in front of screens (Hill et al., 2020).

##### Ethnicity/culture

Studies with American samples have found that Black and Hispanic parents are more likely to expose infants to screens (Hish et al., 2020). According to an American study, Black parents are more likely to perceive the benefits of child screen use (Rideout & Robb, 2020). This same study indicated that there appears to be a widening racial gap, with Hispanic/Latinx and Black children spending increasing amounts of time with screens (Rideout & Robb, 2020). New studies are needed to better understand the cultural factors involved in children’s media use (Jordan & Prendella, 2019). Furthermore, as previously noted, ethnicity and race may further influence how children respond to screens due to the presence of negative stereotypes and a lack of diversity (Adams-Bass et al., 2014). Finally, most research has focused on children from WEIRD (Western, Educated, Industrialized, Rich and Democratic) countries (Jordan & Prendella, 2019). As such, research on screen use by children in the Global South and in countries facing high levels of income inequality is needed.

Little research has examined how child health, fitness, or overweight status may interact with their media habits to contribute to social, cognitive, and physical outcomes. In particular, children with predispositions to poorer health may be more vulnerable to the obesogenic effects of excessive screen time. Similarly, overweight children may be more vulnerable to psychosocial consequences of exposure to contents that can lead to negative social comparisons between themselves and characters depicted as being more socially or physically attractive.

##### Family context

Certain characteristics of parents like maternal age, and lower education, income, and socioeconomic status have been linked to higher levels of child screen time (Bernard et al., 2017; Rideout & Robb, 2020). Furthermore, children from more disadvantaged families appear to be more vulnerable to the negative impacts of screens on cognitive development (Ribner et al., 2017). Finally, as previously explained, parent’s own screen time also appears to be a consistent predictor of child screen time and media use habits (Lauricella et al., 2015; Madigan et al., 2020a, 2020b). Less research has examined parental media literacy and beliefs regarding the educational value of child screen time as predictors and moderators of the impact of media on children. Furthermore, there is much less research examining how fathers or other caretakers of children, such as grandparents, may use screens with young children. There is some research suggesting that grandparents may use screens despite parental objections. For instance, according to a Brazilian study, grandparents were more likely to expose infants to screens than parents (Canani da Rosa et al., 2021).

#### Explaining associations between screen use and developmental outcomes

To advance theory and practice, it is important to better understand what explanatory mechanisms may account for the positive and negative consequences of child screen time. *Displacement hypotheses* state that screen media can take time away from developmentally enriching activities such as real-life interactions, active play, and sleep (Radesky et al., 2015a, 2015b). However, the extent to which the displacement of specific activities contributes to child outcomes remains largely unexamined. Displacement explanations are also rooted in neurobiological theories of early childhood development which recognize the importance of early environment-dependent brain growth. In this sense, the stimulation provided by screens is believed to be of lower quality than that provided by real-life interactions between a child and their caretaker and the physical and sensory environment. In support of this hypothesis, research has found that unhealthy patterns of screen use are associated with less-than-optimal development in brain areas that play a role in executive functions and language (Hutton et al., 2020).

Some of the consequences of child media use may be explained by the physical characteristics of screens. In particular, the blue light emitted by some devices can interfere with melatonin production and may be disruptive to circadian rhythms and sleep. Another possibility is that screen media influences child outcomes by disrupting attentional and executive function systems because it is overstimulating for developing brains (Christakis et al., 2018). In line with the *overstimulation hypothesis*, there is some evidence that screen use may undermine school readiness because of its negative association with executive functions (Ribner et al., 2017). For instance, 12 min of exposure to fast-paced cartoons can undermine executive functions in the short-term (Lillard & Peterson, 2011).

Finally, *social learning mechanisms* are also likely to contribute to positive and negative consequences of digital media use (Greitemeyer & Osswald, 2010). Children learn by observing models around them (Bandura, 1977). In addition to modeling others in their immediate environment, children may also choose to imitate their favorite character from a show or video game. As such, depictions of helping behavior, violence, racial and gender stereotypes, or a lack of diversity and positive representations can come to shape child perceptions and expectations of their social world.

Research highlights that a combination of circumstances, including features of child screen use and the characteristics of viewers contribute to positive and negative child outcomes. The mechanisms linking media use to positive and negative consequences are likely to vary according to the nature of children’s screen usage and outcomes being considered. For instance, the association between media use and unhealthy weight gain may be largely explained by the displacement of exercise and sleep, whereas impacts of media on social skills may be more sensitive to media content and the nature of usage, with prosocial programs and using screens that allow video chatting benefiting social development. Finally, mechanisms are likely to vary according to child characteristics. For instance, infants are likely to be more sensitive to the displacement of developmentally enriching activities, whereas older preschool children may be more sensitive to the social learning processes afforded by media.

## Discussion

### Summary of findings

In summary, our review suggests that exposure to longer amounts of screen time can have a negative impact on preschooler health and development. Certain contents, including those that contain violence, or that are fast-paced, or adult-directed may contribute to additional negative cognitive and psychosocial outcomes for young children. In terms of contexts of screen use, use during mealtimes, before bedtime, or in moments when children are experiencing negative emotions are likely to undermine health and self-regulation development. Considerably fewer studies have examined the positive effects of screen use on child development. Exposure to high-quality educational contents intended for young children was associated with improved social and learning outcomes in preschoolers. Finally, interactive screen use such as joint engagement with an adult appears to benefit child learning. Furthermore, the use of video chat technology was associated with positive learning outcomes in infants and preschoolers.

Some children may also be more vulnerable to the negative impacts of screen use. More specifically, younger children, those presenting more temperamental and behavioral challenges, and those facing higher levels of family adversity may be more likely to experience negative outcomes as a result of their media habits. Finally, many of the negative consequences of screen use on the development of young children could be explained by the *displacement* of important developmental activities or because of the physical characteristics of the screen such as the blue light they emit. In contrast, other negative and positive outcomes may be explained by the type of contents children are engaging with and the nature of their screen use. In particular, exposure to fast-paced programs and videos can be harmful to the development of attention skills. In contrast, high-quality, child-directed educational programs that encourage children to sing, rhyme, and engage with the program, as well as shows that feature a high frequency of prosocial, altruistic behavior, may foster cognitive development and social competence.

### Limitations of existing research

 Several important limitations of existing research should be addressed to help advance knowledge and build theory in this area. First, most research has focused on screen time, as opposed to other features of use such as content, context of use, and nature of use. Furthermore, consequences of screen use are likely to vary according to child characteristics (ex., age, sex,). As such, research that is capable of simultaneously considering multiple dimensions of child screen habits is needed to help clarify which *profiles* or patterns are more closely related to negative, positive, or neutral outcomes. Much of the research on media use by children, families, and schools has been limited by using cross-sectional designs, which make it difficult to rule out reverse causation. As such, longitudinal designs able to account for bidirectional effects and experimental studies are needed. In addition, much of the longitudinal work examining the consequences of media use on children has drawn on samples that grew up prior to 2010, before the explosion in the popularity of tablets and smartphones. Research needs to be expanded to take into consideration newer and evolving devices. Finally, little research has examined the role of families and schools in shaping children’s screen use and their health and developmental consequences. Moreover, research has yet to examine how home- and school-based usage interactively and cumulatively contribute to child developmental outcomes.

### Clinical implications and suggested interventions to reduce harm and promote positive media use

Multidimensional approaches that simultaneously consider screen time duration, content, context, and nature of use can better inform harm-reduction approaches by helping us identify which patterns or profiles of young children’s media use are more closely associated with positive, neutral, and negative outcomes. In addition, to better direct early prevention and intervention efforts, research should systematically pay attention to individual child and family characteristics that could contribute to differential susceptibility. Finally, a better understanding of the different mechanisms (ex., displacement, social learning) that account for negative, positive, or neutral consequences on health and development could also help us better support intervention efforts.

Research suggests that interventions that begin in early childhood are likely to be the most effective for helping children develop and sustain balanced media use habits (Jones et al., 2013; Wahi et al, 2011). As such, education and health practitioners working with families can adopt a harm-reduction approach by informing parents about the uses most closely associated with negative outcomes (ex., excessive screen duration, bedtime use, and use before mealtimes) while also informing them about healthy screen use habits (ex., limiting time to one hour per day, co-viewing with the child, prioritizing high-quality child-directed educational content). Professionals should also encourage families to use a media plan (American Academy of Pediatrics, 2021), which is customizable and can help parents establish healthy media routines as well as screen-free activities. Interventions may also target parents’ own screen use, as parent screen time is a strong predictor of child screen time (Lauricella et al., 2015).

Finally, schools and daycare settings can also contribute to reducing harm from screens. Media-based activities have been found to be common in childcare settings (Christakis & Garrison, 2009). As such, increased efforts could be directed at sensitizing early childhood professionals to the potential risks associated with accumulating intensive screen use in early childhood. Finally, schools and daycare settings are ideally suited to help initiate children to a wide variety of screen-free activities. In particular, schools can work with the greater community to initiate children to a variety of activities that do not involve screens, such as sports, dance, nature exploration, or cooking.

## Conclusion

Our narrative review supports continuing to limit screen media for children under the age of two and encouraging parents to establish a family media plan and to co-view and supervise older preschoolers’ screen use activities. Effective family media plans should take inventory of healthy screen time allocations for each member of the family including parents and should allow for screen-free times such as during mealtimes or during bedtime routines. Collectively, research also supports advocating for screen-free daycare and preschool environments. As such, interventions could also target education and community centers that are involved with the caretaking of young children. From a harm-reduction perspective, selecting screen use activities carefully based on children’s own characteristics (ex., age, interests, behavioral profile) and based on features of the content, nature of use, and context of use may also help parents make selections and choices that maximize benefits and minimize harms from screen use.

## Data Availability

N/A.
