# Correction: Reducing harm and promoting positive media use strategies: new perspectives in understanding the impact of preschooler media use on health and development

**DOI:** 10.1186/s41155-023-00266-y

**Published:** 2023-08-28

**Authors:** Caroline Fitzpatrick, Marie-Andrée Binet, Emma Cristini, Maíra Lopes Almeida, Mathieu Bégin, Giana Bitencourt Frizzo

**Affiliations:** 1 Département de L‘Enseignement Au Préscolaire Et Au Primaire, Université de Sherbrooke, Sherbrooke, Québec Canada; 2 Department of Childhood Education, University Johannesburg, Johannesburg, South Africa; 3 Faculté de Médecine Et Des Sciencesnces de La Santé, Université de Sherbrooke, Sherbrooke, Canada; 4 Department of Psychology, Universidade Federal Do Rio Grande Do Sul, Porto Alegre, Brazil

**Correction:** **Psicol. Refl. Crít. 36, 19 (2023)**

**https://doi.org/10.1186/s41155-023-00262-2**

Following publication of the original article (Fitzpatrick et al., 2023), the authors reported errors in two authors' name, which have been corrected from:

Maira Almeida Lopes to Maíra Lopes Almeida, Giana Frizzo to Giana Bitencourt Frizzo

The original article (Fitzpatrick et al., 2023) has been updated.
